# Community-acquired pneumonia – use of chest x-rays for diagnosis in family practice

**DOI:** 10.1186/s12875-022-01872-y

**Published:** 2022-10-28

**Authors:** Sophia Eilat-Tsanani, Carmel Kasher, Hana Levine-Kremer

**Affiliations:** 1 The Department of Family Medicine, Clalit Health Services, Northern Region, Israel; 2 The Department of Family Medicine, Azrieli Faculty of Medicine, Bar Ilan University, Safed, Israel; 3 The Department of Family Medicine, Dan Petach Tikva Region, Clalit Health Services, Tel Aviv, Israel

**Keywords:** Pneumonia, x-rays, Family practice, Periphery, Accessibility

## Abstract

**Background:**

According to guidelines, the diagnosis of pneumonia should be confirmed by chest x-ray, ensuring appropriate management and wise use of antibiotics. Our study aimed to describe use of x-rays by family doctors and patients following diagnosis of pneumonia in primary care practices in the north of Israel.

**Methods:**

This was a retrospective database study including adults diagnosed with pneumonia, assessing rates of referral and actual use of chest x-rays. We examined rates of referral for chest x-rays and rates of adherence to the referral, according to age, gender, smoking status, comorbidities and distance of residence from the radiology facility.

**Results:**

During one year there were 4,230 diagnosed cases of pneumonia in the practice, of which 2,503 were referred for chest x-rays, and 1,920 adhered to the referral (45% of those diagnosed with pneumonia). The rate of referral was higher when the radiology facility was located in the same city as the family doctor compared to outside the city (69.7% and 53.2%, p < 0.001). Patients aged 40–64 were referred more than patients aged 18–39 or 65+ (61.5% vs. 56.5% and 58.3%, p = 0.03). Actual use of chest x-rays (considering both referral and adherence) was more likely when the radiology facility was in the same health centre or city than when it was outside the city [OR = 2.4; 95% CI: 2.1–2.8]; patients aged 65 + or 40–64 were more likely to adhere to the referral for x-ray than those aged 18–39 [OR = 1.3; 95% CI: 1.1–1.6, OR = 1.2; 95% CI: 1.0–1.4, respectively].

**Conclusion:**

Accessibility of radiology facilities seems to be an important factor associated with both doctors’ decisions and patients’ adherence to the referral for chest x-rays.

**Supplementary Information:**

The online version contains supplementary material available at 10.1186/s12875-022-01872-y.

## Background

Community-acquired pneumonia is an infectious disease with a wide spectrum of presentation, carrying the potential for high morbidity and mortality, particularly in the elderly population and among patients with significant comorbidities [1, 2].

Accurate diagnosis of pneumonia is essential for appropriate care and appropriate use of antibiotics. Clinical diagnosis based on history taking and physical examination is subject to misjudgement due to lower levels of accuracy (74% sensitivity, 84% specificity [3]). This may lead to over- or underuse of antibiotics [4].

Chest x-ray is the recommended examination for diagnosis of pneumonia, according to guidelines [5] and regularly used textbooks, [6–9] which prescribe chest radiograph as an obvious component of the pneumonia diagnostic process. However, in contrast to the conditions in emergency departments, chest x-ray examination is not always accessible in the community.

A Cochrane review [10] aimed to evaluate the effectiveness of chest radiographs in addition to clinical judgement, compared to clinical judgement alone, in the management of acute lower respiratory infections. It concluded that there is no difference in the outcome of pneumonia detected with or without actual use of a chest x-ray, but there remains a concern of over-prescription of antibiotics.

Our study aimed to assess compliance with guidelines for use of chest x-rays in the diagnosis of community acquired pneumonia, according to doctors’ referral during the visit and patients’ adherence thereafter.

## Methods

This was an observational database study. The study population comprised patients above 18 years of age who were under the care of Clalit Health Services (CHS).

CHS is one of four health maintenance organizations in Israel which people may choose for their medical care within the national health insurance scheme. People are registered with their family physician, [11] to whom visits are free of charge. Similarly, blood tests are free of charge while x-ray examination carries a small fee. The immediate tests available for use in the practice are ECG and urinalysis. Blood tests are transferred to the laboratories and results can be retrieved within 24 h.

The study region is the northern periphery of Israel, where CHS provides care for 583,000 people, over 70% of the region’s residents. In the northern region care is provided to CHS patients in 250 clinics, of which 160 are rural clinics and the remainder are urban health centres and medium-sized primary care clinics. x-rays are performed in health centres or in designated radiology facilities. Interpretation of the chest X-ray is obtained within 24 h.

CHS operates an integrated electronic medical and administrative file for each patient, based on the International Classification of Diseases (9th Revision). Chronic diseases that take part in the Quality Measures program, such as type 2 diabetes, cardiovascular diseases, asthma, and chronic obstructive pulmonary disease (COPD), are also cross-validated against medication possession records and laboratory data through an automated disease-specific process [12, 13].

The study population comprised cases where patients visited their family doctors over the course of one year in 2015, with a visit diagnosis of pneumonia. The index visit was defined as the first in a six-week period with pneumonia in the diagnosis field. Information on the interpretation of the x-ray was retrieved during a 14-day period from the day of the index visit. As a result, without having other measures, the diagnosis of pneumonia was clinical, based on the patient’s history and findings in the physical examination. All subsequent visits with the same diagnosis during a six-week period, as well as visits following hospitalization, visits to the emergency department, and chest x-rays performed later than 14 days from the index visit, were omitted from the study.

Independent demographic variables included patient gender and age. Accessibility of radiology facilities was defined by their location: either in the same health centre and city as the family doctor, or outside the city. Variables of chronic comorbidities included diabetes, COPD, asthma, ischemic heart disease and heart failure, and current or past smoking.

The outcome measure was a referral for a chest x-ray. We compared cases that were or were not referred for a chest x-ray. Among cases that were referred for a chest x-ray, we compared adherence vs. non-adherence to the referral.

### Statistical analyses

The data were analysed by SAS version 9.4. Categorical data were reported as percentages (%). Association with referral for a chest x-ray or adherence with the referral for a chest x-ray was performed using the Chi-square test. A logistic regression model was designed to examine the prediction of actual use of a chest x-ray (for all pneumonia patients), taking into account demographic and morbidity variables. P-values of less than 0.05 were considered significant.

## Results

We followed 4,230 cases that were eligible for the study because the visit was terminated with a diagnosis of pneumonia during one year. The study sample contained a high proportion of patients over 40 years of age (75.2%). Radiology facilities were located more often outside the city where the family doctor’s practice is located (63.8%). A diagnosis of at least one of the listed chronic comorbidities was reported in 24.4% of those patients (Table 1).

**Table 1:** Demographics and morbidity characteristics of the study participants

Variables		N	(%)
Gender	Men	2064	(48.8)
	Women	2166	(51.2)
Age	18–39	1047	(24.8)
	40–64	1702	(40.2)
	65 +	1481	(35.0)
Distance to the radiology facility	Out of the city	2699	(63.8)
	In the clinic or in the city	1531	(36.2)
Smoking	Never	3535	(83.6)
	Past or current	695	(16.4)
Asthma	No	3893	(92.0)
	Yes	337	(8.0)
CHF	No	4176	(98.7)
	Yes	54	(1.3)
COPD	No	4086	(96.6)
	Yes	144	(3.4)
Diabetes	No	3775	(89.2)
	Yes	455	(10.8)
IHD	No	3848	(91.0)
	Yes	382	(9.0)
Chronic comorbidities	None	3198	(75.6)
	At least one	1032	(24.4)

CHF = congestive heart failure; COPD = chronic obstructive pulmonary disease; IHD = ischemic heart disease

**Fig. 1:**
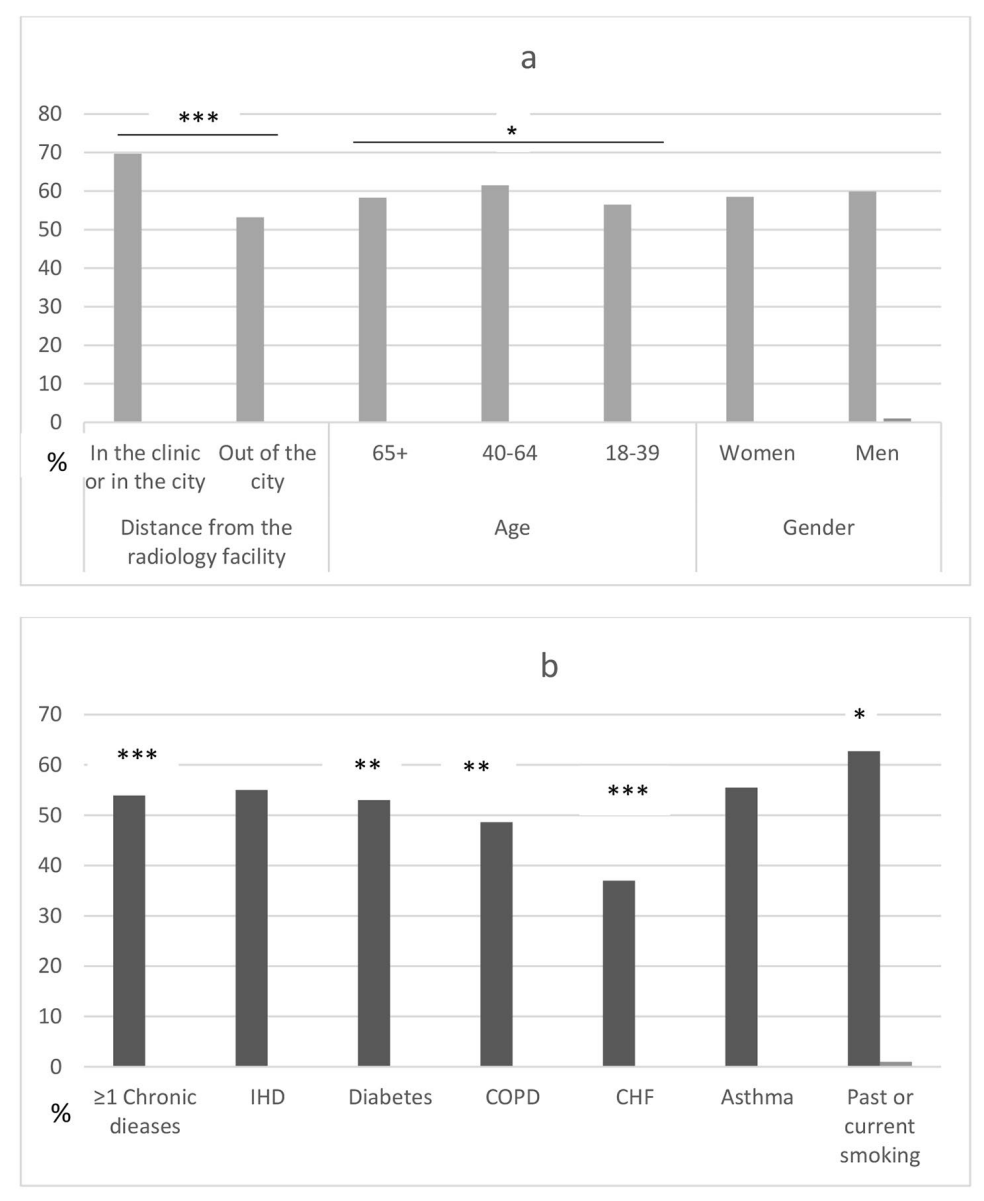
Referral for chest x-ray in patients with visit diagnosis of pneumonia: (a) association with demographic characteristics; (b) association with morbidity characteristics. * p < 0.05, ** p < 0.01, *** p < 0.001

Referrals for chest x-rays were reported in 2,503 cases of diagnosis with pneumonia (59.2%). A higher rate of referral was reported in patients aged 40–64 than in patients aged 18–39 or 65 years and older, and in patients who were referred to a radiology facility in the same health centre or city compared to a facility outside the city (Fig. 1a). Rate of referral was higher in smokers than non-smokers. In patients with comorbidities, the rate of referral was not higher, but rather lower, than in patients without comorbidities (Fig. 1b).

**Fig. 2:**
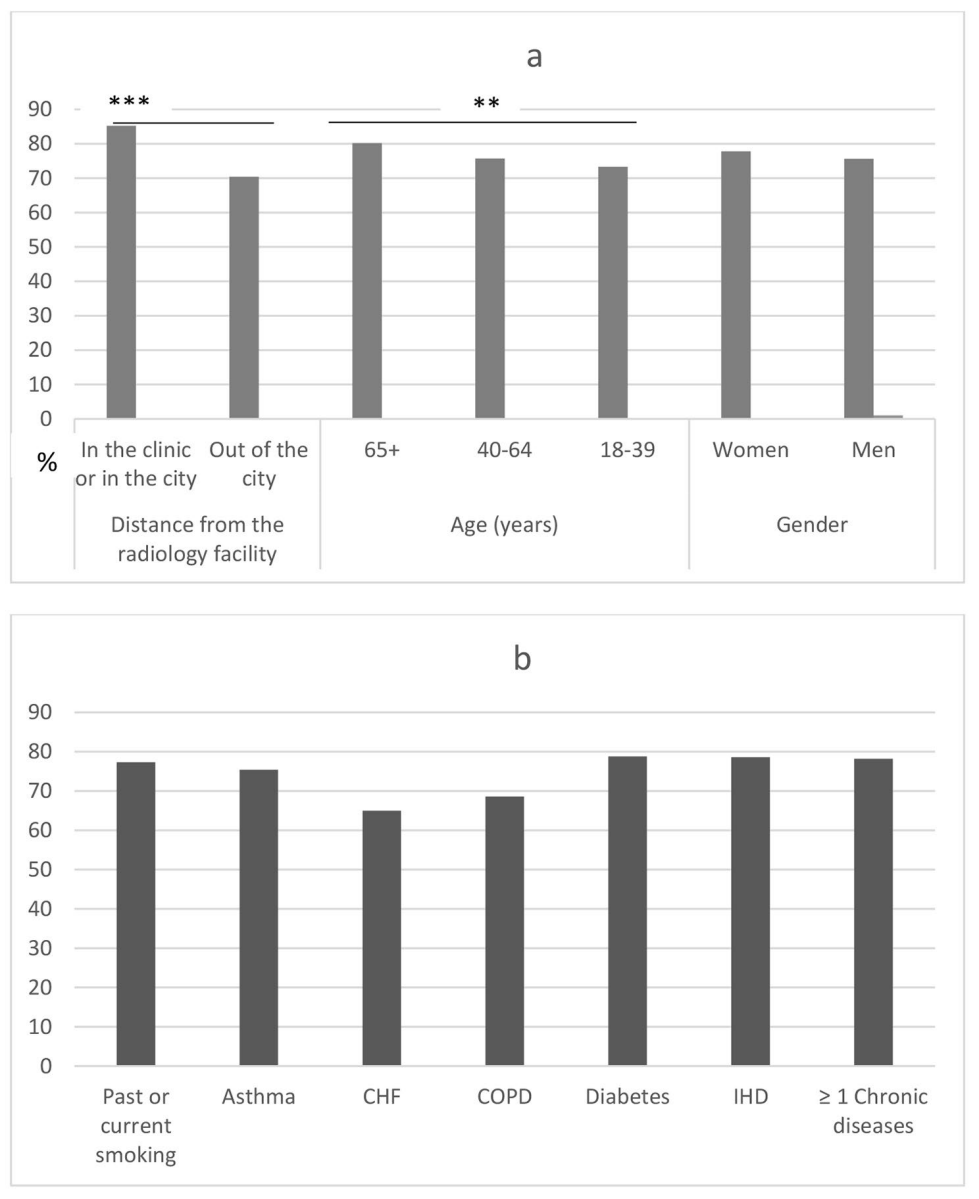
Adherence to referral for chest x-rays in patients with a visit diagnosis of pneumonia: (a) association with demographic characteristics; (b) association with morbidity characteristics. * p < 0.05, ** p < 0.01, *** p < 0.001

Adherence to the referral for a chest x-ray was detected in 1,920 (76.7%) of the cases that were referred for chest x-rays. Within this group of patients, a higher rate was observed in patients aged 65 and older than in younger age groups, and in patients who were referred to a radiology facility in the same health centre or city than in those referred to a facility outside the city (Fig. 2a). Rates of adherence were similar across health variables (Fig. 2b).

As mentioned above, chest x-rays were used in 1,920 cases (45.4% of patients with a diagnosis of pneumonia during the visit). We fitted a model to predict the actual use of chest x-rays. The model included 4,230 cases: 1,920 that were referred and adhered to the referral, vs. 1,727 that were not referred and did not undergo a chest x-ray and 583 that were referred but did not adhere to the referral .

Actual use of chest x-rays was higher among patients who were referred to a radiology facility in the same health centre or city than in those referred to a facility outside the city [OR = 2.4; 95% CI: 2.1–2.8]; it was also higher in patients aged 65 and older, and in those aged 40–64, than in those below the age of 40 [OR = 1.3; 95% CI: 1.1–1.6, OR = 1.2; 95% CI: 1.0–1.4, respectively]. Having any chronic disease was negatively associated with actual use of a chest x-ray (Table 2).

**Table 2:** Odds ratio of actual use of a chest x-ray following a visit diagnosis of pneumonia (whether referred or not referred)

Variable		OR (95% CI)
Gender	Men	1.00
	Women	0.95 (0.84–1.08)
Age (years)	18–39	1.00
	40–64	1.20 (1.02–1.41)*
	65+	1.30 (1.09–1.55) **
Distance to the radiology facility	Out of the city	1.00
	In the clinic or in the city	2.44 (2.14–2.77) ***
Smoking	Never	1.00
	Past or current	1.08 (0.91–1.28)
Chronic comorbidities	None	1.00
	At least one	0.74 (0.63–0.87)***

The model included 4,230 patients, 45.4% of whom performed chest x-rays

* p-value < 0.05, ** p-value < 0.01, ***p-value < 0.001

## Discussion

Our study brings evidence from real life data demonstrating only partial compliance with guidelines for diagnosing pneumonia. Specifically, we have provided evidence on the underuse of chest x-rays for confirming the diagnosis of pneumonia by family doctors. Less than half of the patients who received a pneumonia diagnosis from their family doctor underwent a chest x-ray. Family doctors referred 60% of cases with pneumonia as the visit diagnosis; 78% of them adhered to the referral.

Accessibility of the radiology facility seems to be a major factor contributing to actual use of chest x-rays, associated both with referral by family doctors and adherence by their patients. Another predicting factor was older age – people older than 65 adhered more than others to the referral for a chest x-ray. Chronic comorbidity was not associated with actual use of chest x-rays, even with respect to diseases more associated with pulmonary morbidity, such as asthma and COPD.

The northern district of CHS is spread over a large area in the periphery of Israel. Care is provided through many clinics varying in size from rural villages to urban health centres. Radiology facilities can be located in the same health center as the family doctor or away from the primary care clinic, necessitating a special drive after the visit to the family doctor that can last up to an hour. Patients make their own arrangements for transportation, often by private car. This infrastructure may influence the family doctors’ decision and their patients’ adherence. The higher rate of adherence in patients aged 65 and older to the referral for a chest x-ray can be explained by acknowledgement of the threat of pneumonia at older age.

In the absence of radiology evidence for pneumonia, the medical decision is empirical and based on evidence with weak validity. We assume that diagnosis of pneumonia is linked to antibiotic treatment. According to our study outcomes, more than half of the patients were managed without radiological evidence, possibly with antibiotics [4, 14].

The added value of x-rays in the diagnostic process for pneumonia was evaluated in a study^8^ conducted both in primary care clinics and a hospital emergency department; it reported 74% sensitivity and 84% specificity for clinical diagnosis without chest x-rays, but only 27% positive predictive value. Hopstaken et al.^14^ demonstrated that diagnosis of pneumonia based on history taking and physical examination alone led to misjudgement and misuse of antibiotics, reflected in 86% overuse and 16% underuse. Another study showed that 20% of patients who presented to primary care clinics [4] with cough and fever had x-ray findings compatible with pneumonia; when the diagnosis was based on clinical judgement alone the rate of antibiotic prescription was twice as high.

Given the barriers set by long distances in the periphery, other options for confirming a bedside diagnosis of pneumonia should be considered. A systematic and meta-review found that clinical features such as respiratory rate > 20/min, temperature ≥ 38 °C, pulse rate > 100/min and crackles showed the best pooled positive likelihood for pneumonia [15]. Another study conducted in primary care clinics recorded the outcome of visits of patients suspected for pneumonia according to doctors’ suspicions based on findings in physical examinations and results of blood tests, compared to the outcome of chest x-rays. In this study, the results of c-reactive protein (CRP) blood tests contributed more than physical examination parameters to the diagnosis of pneumonia [16]. A Cochrane review from 2014 [17] evaluated the contribution of the point of care (CRP) test for appropriate use of antibiotics for pneumonia. The authors concluded that the CRP test could assist in clinical diagnosis. The review included studies that did not necessarily use x-rays. The use of point of care ultrasound for diagnosis is a promising tool, but it is still not in sufficiently wide use due to training and cost limitations [16–19].

### Strengths and limitations

Our study brings evidence from a comprehensive database with high validity of doctors’ activities and patients’ performance. However, our study is limited by missing variables indicating the clinical situation that could influence the doctors’ judgement, such as indications for severity of the disease. Similarly, we did not have the results of the chest x-rays, so we cannot discuss the implications for patient care. We also can only assume antibiotic use, since it was outside the aims and scope of our study.

## Conclusion

According to guidelines, clinical diagnosis of pneumonia should be confirmed by chest x-ray. In practice, more patients are treated without radiological evidence of pneumonia. Accessibility of radiology facilities appears to be an important contributing factor for both doctors’ and patients’ decisions. This indicates a need to develop other measures to confirm or at least rule out the diagnosis of pneumonia according to the severity of the condition, together with improving accessibility to radiology facilities.

## Electronic supplementary material

Below is the link to the electronic supplementary material.

Supplementary Material 1

## Data Availability

The datasets generated and/or analyzed during the current study contain patient-level data and are not publicly available due to the privacy regulations of CHS.
