# Supplementary material for: Community-acquired pneumonia – use of chest x-rays for diagnosis in family practice
Source: BMC Prim Care. 2022 Oct 28;23:271. doi: 10.1186/s12875-022-01872-y (PMC9615378; doi:10.1186/s12875-022-01872-y)
Supplement: Supplementary file 1 — Supplementary Material 1 [file 12875_2022_1872_MOESM1_ESM.docx]

**Supplementary data**

**Table S1:** Demographics and morbidity characteristics of patients with visit diagnosis of pneumonia and referral for chest x-ray

| Variables | | | | Were not referred for chest x-ray examination | | | | Referred for chest x-ray examination | | | | Significance^-^ | |
| --- | --- | --- | --- | --- | --- | --- | --- | --- | --- | --- | --- | --- | --- |
|  |  |  |  | N (%) | |  | | N (%) | |  | |  |  |
| Total (N=4,230) | | | | 1727 (40.8) | |  | | 2503 (59.2) | |  | | - | |
| Gender | | Men | | 828 (40.1) | |  | | 1236 (59.9) | |  | | 0.3583 | |
|  |  | Women | | 899 (41.5) | |  | | 1267 (58.5) | |  | |  |  |
| Age | | 18-39 | | 455 (43.5) | |  | | 592 (56.5) | |  | | 0.0261 | |
|  |  | 40-64 | | 655 (38.5) | |  | | 1047 (61.5) | |  | |  |  |
|  |  | 65 + | | 617 (41.7) | |  | | 864 (58.3) | |  | |  |  |
| Distance from the radiology facility | | Out of the city | | 1263 (46.8) | |  | | 1436 (53.2) | |  | | <0.0001 | |
|  |  | In the clinic or in the city | | 464 (30.3) | |  | | 1067 (69.7) | |  | |  |  |
| Smoking | | Never | | 1468 (41.5) | |  | | 2067 (58.5) | |  | | 0.0367 | |
|  |  | Past or current | | 259 (38.3) | |  | | 436 (62.7) | |  | |  |  |
| Asthma | | No | | 1577 (40.5) | |  | | 2316 (59.5) | |  | | 0.1516 | |
|  |  | Yes | | 150 (44.5) | |  | | 187 (55.5) | |  | |  |  |
| CHF | | No | | 1693 (40.5) | |  | | 2486 (59.5) | |  | | 0.0009 | |
|  |  | Yes | | 34 (63.0) | |  | | 20 (37.0) | |  | |  |  |
| COPD | | No | | 1653 (40.5) | |  | | 2433 (59.5) | |  | | 0.0087 | |
|  |  | Yes | | 74 (51.4) | |  | | 70 (48.6) | |  | |  |  |
| Diabetes | | No | | 1513 (40.1) | |  | | 2262 (59.9) | |  | | 0.0044 | |
|  |  | Yes | | 214 (47.0) | |  | | 241(53.0) | |  | |  |  |
| IHD | | No | | 1555 (40.4) | |  | | 2293 (59.6) | |  | | 0.0800 | |
|  |  | Yes | | 172 (45.0) | |  | | 210 (55.0) | |  | |  |  |
| Chronic comorbidities | | None | | 1251(39.1) | |  | | 1947 (60.9) | |  | | 0.0001> | |
|  |  | At least one | | 476 (46.1) | |  | | 556 (53.9) | |  | |  |  |

CHF=congestive heart failure; COPD= chronic obstructive pulmonary disease; IHD=ischemic heart disease

**Table S2:** Demographics and morbidity characteristics of patients with visit diagnosis of pneumonia who do or do not adhere to the referral for a chest x-ray

| Variables | | Non-adherence to referral for chest x-ray examination | Adherence to referral for chest x-ray examination | Significance |
| --- | --- | --- | --- | --- |
|  |  | N ((% | N (%) |  |
| Total | | 583 (23.3) | 1920 (76.7) | - |
| Gender | Men | 302 (24.4) | 934 (75.6) | 0.1820 |
|  | Women | 281 (22.2) | 986 (77.8) |  |
| Age (years) | 18-39 | 158 (26.7) | 434 (73.3) | 0.0058 |
|  | 40-64 | 254 (24.3) | 793 (75.7) |  |
|  | 65 +a | 171 (19.8) | 693 (80.2) |  |
| Distance from the radiologic facility | Out of the city | 425 (29.6) | 1011 (70.4) | 0.0001> |
|  | In the clinic or in the city | 158 (14.8) | 909 (85.2) |  |
| Smoking | Never | 484 (23.4) | 1583 (76.6) | 0.7502 |
|  | Past or current | 99 (22.7) | 337 (77.3) |  |
| Asthma | No | 537 (23.2) | 1779 (76.8) | 0.6603 |
|  | Yes | 46 (24.6) | 141 (75.4) |  |
| CHF | No | 576 (23.2) | 1907 (76.8) | 0.2842 |
|  | Yes | 7 (35.0) | 13 (65.0) |  |
| COPD | No | 561 (23.1) | 1872 (76.9) | 0.1024 |
|  | Yes | 22 (31.4) | 48 (68.6) |  |
| Diabetes | No | 532 (23.5) | 1730 (76.5) | 0.4105 |
|  | Yes | 51 (21.2) | 190 (78.8) |  |
| IHD | No | 538 (23.5) | 1755 (76.5) | 0.5045 |
|  | Yes | 45 (21.4) | 165 (78.6) |  |
| Chronic comorbidities | None | 462 (23.7) | 1485 (76.3) | 0.3334 |
|  | At least one | 121 (21.8) | 435 (78.2) |  |

CHF= congestive heart failure; COPD=chronic obstructive pulmonary disease; IHD=ischemic heart disease
